# Influence of Intestinal Microbiota Transplantation and NleH Expression on *Citrobacter rodentium* Colonization of Mice

**DOI:** 10.3390/pathogens7020035

**Published:** 2018-03-30

**Authors:** Gaochan Wang, Leigh Ann Feuerbacher, Philip R. Hardwidge

**Affiliations:** Department of Diagnostic Medicine/Pathobiology, Kansas State University, Manhattan, KS 66506, USA; gaochan.wang@gmail.com (G.W.); feuerbac@vet.k-state.edu (L.A.F.)

**Keywords:** *Citrobacter*, colonization, microbiota

## Abstract

The intestinal microbiota plays an important role in regulating host resistance to enteric pathogens. The relative abundance of the microbiota is dependent upon both genetic and environmental factors. The attaching and effacing pathogens enteropathogenic *Escherichia coli*, enterohemorrhagic *E. coli*, and *Citrobacter rodentium* cause diarrheal disease and translocate type III secretion system effector proteins into host cells to inhibit pro-inflammatory host responses. Here we determined the influence of both the intestinal microbiota and the expression of the *C. rodentium* NleH effector on *C. rodentium* colonization in different mouse models. We performed fecal transplantation experiments between C57BL/6J and C57BL/10ScNJ mice and found that such microbiota transfers altered both the host resistance to *C. rodentium* infection as well as the benefit or detriment of expressing NleH to *C. rodentium* intestinal colonization.

## 1. Introduction

Enteropathogenic *Escherichia coli* (EPEC), enterohemorrhagic *Escherichia coli* (EHEC), and *Citrobacter rodentium* are attaching and effacing (A/E) bacterial pathogens that cause infectious diarrhea [1]. EPEC and EHEC are human pathogens, while *C. rodentium* colonizes mice and is used as a model organism for in vivo studies of A/E pathogens [2]. These organisms use a type III secretion system to translocate effector proteins into host cells [3]. Some of these effectors inhibit the activation of host pro-inflammatory responses to infection by targeting regulatory components of the NF-kB signaling pathway [4].

We characterized the activity of the NleH effector and its role in *C. rodentium* virulence [5,6,7,8]. NleH inhibits the NF-kB pathway by preventing the phosphorylation of the ribosomal protein S3 (RPS3) via the inhibitor kB kinase beta (IKKβ) [8]. RPS3 has a moonlighting function as an NF-kB subunit that guides the NF-kB complex to specific promoters and thus plays an important role in transcriptional activation [9]. RPS3 must be phosphorylated on serine 209 and NleH inhibits this phosphorylation and thus reduces RPS3 nuclear translocation [8].

Both C57BL/6J and C57BL/10ScNJ mice have been used to study the role of Toll-like receptor 4 (TLR4) in host responses to *C. rodentium* infection [10]. We previously observed that *C. rodentium* colonization of C57BL/6J mice was reduced ~50-fold upon deleting NleH [5]. When we infected C57BL/10ScNJ mice, which differ from C57BL/6J mice because they have a deletion of the *Tlr4* gene and are thus not responsive to bacterial lipopolysaccharide (LPS), we observed that expressing NleH was detrimental to *C. rodentium* colonization [5]. Host inflammation appeared to contribute to this phenotype because pre-treating C57BL/10ScNJ mice with dextran sodium sulfate, which induces acute colitis in mice [11], restored the expected benefit of expressing NleH to *C. rodentium* [5].

The intestinal microbiota promotes the maturation of immune cells and the development of immunity [12]. Germ-free mice have reduced levels of secretory IgA, defects in the development of gut-associated lymphoid tissues, as well as smaller Peyer’s patches and mesenteric lymph nodes [13]. Germ-free mice are more susceptible to enteric pathogens such as *Shigella flexneri*, *C. rodentium*, *Listeria monocytogenes*, and *Salmonella enterica* serovar Typhimurium [14]. Gut microbiota-mediated control of enteric pathogens is attributed to direct inhibition, barrier maintenance, immune modulation, and altered metabolism. The transplantation of gut microbiota from C3H/HeJ mice that are susceptible to *C. rodentium* infection induces a similar susceptibility to NIH Swiss mice that are otherwise resistant to *C. rodentium* infection by affecting the production of interleukin 22 (IL-22) and antimicrobial peptides [15]. 

Here we sought to determine both the extent to which the intestinal microbiota differ between C57BL/6J and C57BL/10ScNJ mice and whether these differences account for the previously observed [5] benefit or detriment of *C. rodentium* expressing NleH in these infection models.

## 2. Results

### 2.1. C. rodentium Colonization Phenotypes

**Figure 1:**
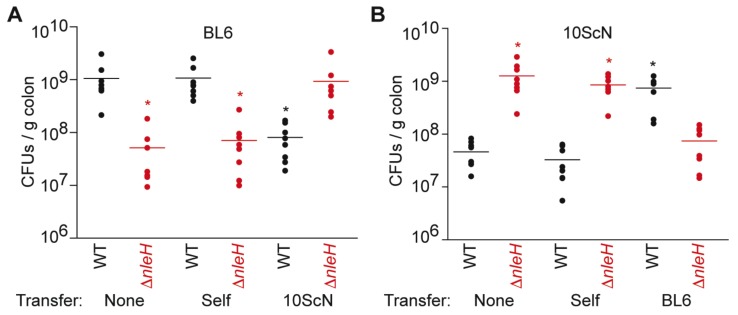
*C. rodentium* colonization as a function of fecal transplantation. (**A**) *C. rodentium* colonization of C57BL/6J mice 14 days post-infection. Where indicated, mice were first transplanted with fecal extracts from either C57BL/6J (self) or C57BL/10ScNJ (10ScN) mice; (**B**) *C. rodentium* colonization of C57BL/10ScNJ mice 14 days post-infection. Where indicated, mice were first transplanted with fecal extracts from either C57BL/10ScNJ (self) or C57BL/6J (BL6) mice. Asterisks indicate significantly different colonization magnitude (Kruskal-Wallis test; *p* < 0.05) as compared with wild-type (WT) *C. rodentium* colonization without fecal transplantation.

We first quantified the abundance of wild-type (WT) and Δ*nleH C. rodentium* in C57BL/6J mice 14 days after infection. As expected from previous studies [5,7], the colonization of Δ*nleH* (5.2 ± 2.0 × 10^7^ CFUs/g colon) was significantly less than that of WT (1.1 + 0.3 × 10^9^ CFUs/g colon) (Figure 1A). We observed the opposite phenotype, as expected from previous studies [5], when we infected C57BL/10ScNJ mice. In C57BL/10ScNJ mice, the colonization of Δ*nleH* (1.3 ± 0.3 × 10^9^ CFUs/g colon) was significantly greater than that of WT (4.6 ± 0.8 × 10^7^ CFUs/g colon) (Figure 1B). Thus, while expressing NleH is beneficial to *C. rodentium* colonization of C57BL/6J mice, it is detrimental to *C. rodentium* colonization of C57BL/10ScNJ mice.

To determine if the mouse intestinal microbiota plays a role in conferring this phenotype, we performed fecal transplantation experiments. We observed that while transferring microbiota within identical strains of mice had no impact on *C. rodentium* colonization, transferring microbiota between differing strains of mice reversed the colonization phenotypes between WT and Δ*nleH C. rodentium* (Figure 1). 

### 2.2. Fecal Transplantation

To determine the extent to which fecal transplantation affected the microbiota composition of recipient mice, we used 16S rDNA sequencing to determine the relative abundance of the microbial communities [16]. We then focused our analyses on the five most abundant bacterial families: Porphyromonadaceae, Prevotellaceae, Rikenellaceae, Lachnospiraceae, and Ruminococcaceae. The Prevotellaceae are anaerobic Gram-negative rods that have been isolated from human feces [17]. There is an increased abundance of *Prevotella* spp. in the mucosa-associated bacteria from patients with active ulcerative colitis compared to those without inflammatory bowel disease [18]. The Porphyromonadaceae are components of the gastrointestinal tract whose abundance has been associated with susceptibility to *Salmonella*-induced colitis [19] and potential resistance to *Clostridium difficile* infection [20]. Rikenellaceae are anaerobic Gram-negative rods that are increased in abundance upon *Listeria monocytogenes* infection [21]. The Lachnospiraceae are anaerobic bacteria that are found in human and animal digestive tracts [22]. Pre-colonization of germ-free mice with murine Lachnospiraceae are protective against *C. difficile* colonization and colonic histopathology [23]. Depletion of Ruminococcaceae has also been associated with nosocomial diarrhea and *C. difficile* infection [24].

**Figure 2:**
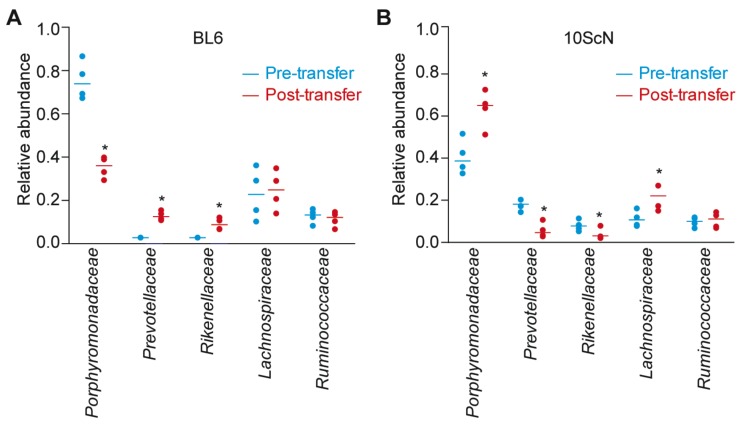
Relative abundance of indicated bacterial families as deduced from 16S rDNA sequence annotation in C57BL/6J (**A**) vs. C57BL/10ScNJ mice (**B**). Asterisks indicate significantly different bacterial family abundance (Kruskal-Wallis test; *p* < 0.05) as a function of transplantation of fecal contents from heterologous mouse strains.

Fecal transplantation from C57BL/10ScNJ mice to C57BL/6J mice resulted in a significant decrease in the abundance of Porphyromonadaceae and a significant increase in the abundance of Prevotellaceae and Rikenellaceae (Figure 2A). Fecal transplantation from C57BL/6J mice to C57BL/10ScNJ mice resulted in a significant increase in the abundance of Porphyromonadaceae and Lachnospiraceae and a significant decrease in the abundance of Prevotellaceae and Rikenellaceae (Figure 2B). 

### 2.3. Microbiota Composition Shifts after C. rodentium Infection of C57BL/10ScNJ Mice 

**Figure 3:**
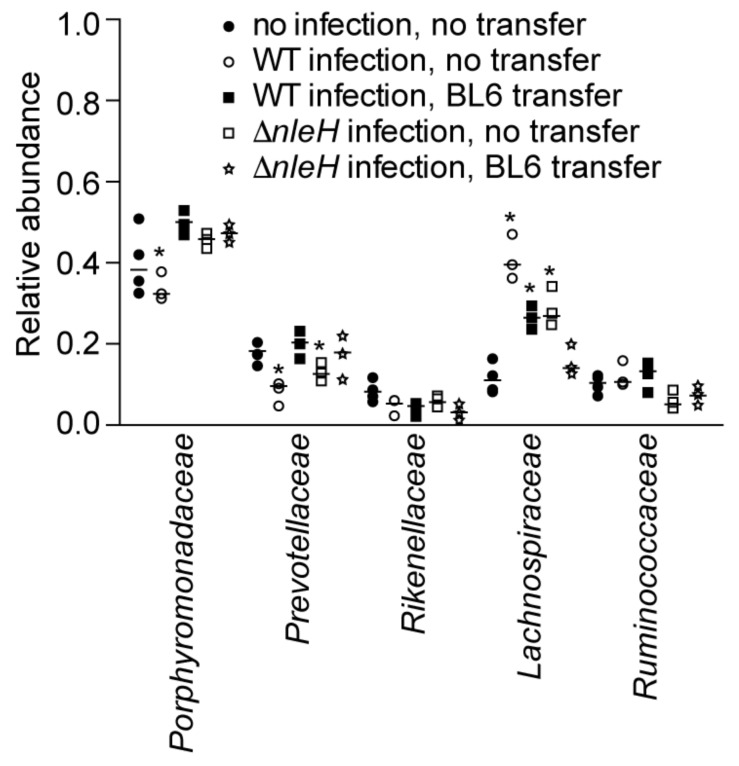
Relative abundance of indicated bacterial families as deduced from 16S rDNA sequence annotation of C57BL6/10ScNJ mice as a function of *C. rodentium* infection and fecal transplantation from C56BL/6J mice. Asterisks indicate significantly different bacterial family abundance (Kruskal-Wallis test; *p* < 0.05) as compared to uninfected C57BL6/10ScNJ mice.

WT *C. rodentium* infection of C57BL/10ScNJ mice resulted in a decreased abundance of Porphyromonadaceae and Prevotellaceae and an increased abundance of Lachnospiraceae, as compared to uninfected mice. By contrast, Δ*nleH C. rodentium* infection resulted in a decreased abundance only of Prevotellaceae and an increased abundance of Lachnospiraceae (Figure 3, blue and red symbols). Prior fecal transplantation from C57BL/6J mice followed by WT *C. rodentium* infection of C57BL/10ScNJ mice resulted in an increased abundance of Lachnospiraceae. By contrast, prior fecal transplantation from C57BL/6J mice followed by Δ*nleH C. rodentium* infection resulted in no significant changes to the relative abundance of the major bacterial families in infected C57BL/10ScNJ mice (Figure 3, green and black symbols). 

## 3. Conclusions

We used 16S rDNA sequencing to address the composition shifts of mouse intestinal microbiomes in response to *C. rodentium* infection. We found that the Bacteroidetes and Firmicutes phyla dominated the intestinal microbiome compositions, consistent with previous findings [15]. The composition of gut microbiota varied substantially among individual mice, but major differences among bacterial families were observed between C57BL/6J and C57BL/10ScNJ mice.

Transplantation of gut microbiota from strains of mice that are susceptible to *C. rodentium* infection induces a similar susceptibility in mice that were previously resistant to *C. rodentium* infection [15]. We found that transferring the microbiota of C57BL/6J mice into C57BL/10ScNJ mice indeed altered the host susceptibility to *C. rodentium* infection and reversed the colonization phenotypes that are dependent upon NleH expression. We observed greater colonization of the *C. rodentium* Δ*nleH* mutant rather than the WT *C. rodentium* in C57BL/10ScNJ mice after fecal transplantation. These data reinforce the notion that NleH expression can have beneficial or detrimental impact to *C. rodentium*, depending upon the relative abundance of specific intestinal microbiota components. Our studies were limited to family-level resolution of the intestinal microbiota. Higher resolution analysis of bacteria genera that differ in abundance after *C. rodentium* infection may permit testing causal relationships between the endogenous microflora and resistance to *C. rodentium*.

## 4. Materials and Methods

### 4.1. Ethics Statement

All animal experiments were performed in strict accordance with the guidelines of Institutional Animal Care and Use Committee at Kansas State University under protocol #3323. This institution complies with all applicable provisions of the Animal Welfare Act and other federal statutes and regulations relating to animals.

### 4.2. Mice and Fecal Transplantations

Three to four-week-old female C57BL/6J and C57BL/10ScNJ mice were obtained from Jackson Laboratory (Bar Harbor, ME, USA). All mice were housed in sterilized cages and fed autoclaved food and water under specific-pathogen-free, controlled temperature, and controlled photoperiod conditions. Fecal transplants were performed as previously described [15]. The native microbiota was depleted by treating mice with a single oral dose (20 mg/mouse) of streptomycin 24 h prior to the first fecal transplantation. Fresh fecal pellets from three to four donor mice were collected and placed in 1 mL transfer buffer (pre-reduced sterile phosphate buffered saline containing 0.05% cysteine HCl) on ice. Fecal pellets were homogenized and centrifuged at 800× *g* for 2 min and the supernatant was collected and diluted (1:3) in transfer buffer. One hundred microliters of diluted fecal supernatant was introduced into recipient mice by oral gavage on six separate occasions, every 48 h. 

### 4.3. C. rodentium Infections

Wild-type and Δ*nleH C. rodentium* DBS100 cultures were grown overnight in LB broth and aerobically cultured with shaking (200 rpm/min) overnight at 37 °C. The cultures were diluted (1:100) into 200 mL LB broth and aerobically cultured with shaking (200 rpm/min) overnight at 37 °C. Cells were harvested by centrifugation at 3000× *g* for 15 min at 4 °C and washed three times with 20 mL of ice-cold PBS. Cells were resuspended into 2 mL of ice-cold 1× PBS and 100-µL aliquots were used to infect mice by oral gavage 2 days after the last microbiota transfer (D14). The actual infection dose was determined by plating 100-µL aliquots of successive dilutions (10^−1^–10^−8^) on LB agar. Mice were monitored twice daily for clinical signs of illness (dehydration, rectal prolapse, loss of responsiveness to stimulation, and >20% weight loss). Colon samples (approximately 4 cm) were collected and stored on ice at necropsy. Feces were removed before weighing tissue. Colon samples were homogenized in PBS, serially diluted, and plated onto MacConkey agar for 24 h at 37 °C. 

### 4.4. Fecal DNA Extraction

Three or four fecal pellets from each mouse were collected on the day of first microbiota transfer (day 1), just prior to infection (day 14), and at euthanasia (day 28). DNA was extracted from fecal pellets by using QIAamp DNA Stool Mini Kits (Qiagen, Venlo, The Netherlands). DNA concentrations were determined by using a Nanodrop 2000 (Fisher Scientific, Waltham, MA, USA). 

### 4.5. Microbial Community Analysis

Microbial community profiles were assessed by using 16S ribosomal DNA sequencing (Cofactor Genomics, St. Louis, MO, USA). Genomic DNA samples from stool samples were used to perform Ion Torrent 16S Ribosomal amplicon library construction. Bacterial Microbial 16S rRNA amplicons were generated via amplification of the V4 hypervariable region of the 16S rRNA gene using single-indexed universal primers. Approximately 40,000 reads per sample were obtained in 400 bp reads. Informatics analysis was performed as described previously [25,26], with contiguous sequences assigned to operational taxonomic units (OTUs) via de novo clustering with a criterion of 97% nucleotide identity [25].

### 4.6. Statistical Analyses

Colonization data and bacterial family abundance data were analyzed using Kruskal-Wallis tests, with *p*-values < 0.05 considered significant.

## References

[1] Kaper J.B. (2005). Pathogenic *Escherichia coli*. Int. J. Med. Microbiol. IJMM.

[2] Deng W., Li Y., Vallance B.A., Finlay B.B. (2001). Locus of enterocyte effacement from *Citrobacter rodentium*: Sequence analysis and evidence for horizontal transfer among attaching and effacing pathogens. Infect. Immun..

[3] Furniss R.C.D., Clements A. (2018). Regulation of the locus of enterocyte effacement in attaching and effacing pathogens. J. Bacteriol..

[4] Gao X., Wang X., Pham T.H., Feuerbacher L.A., Lubos M.L., Huang M., Olsen R., Mushegian A., Slawson C., Hardwidge P.R. (2013). Nleb, a bacterial effector with *Glycosyltransferase* activity, targets GAPDH function to inhibit NF-kappaB activation. Cell Host Microbe.

[5] Feuerbacher L.A., Hardwidge P.R. (2014). Influence of NleH effector expression, host genetics, and inflammation on *Citrobacter rodentium* colonization of mice. Microbes Infect. Inst. Pasteur.

[6] Gao X., Wan F., Mateo K., Callegari E., Wang D., Deng W., Puente J., Li F., Chaussee M.S., Finlay B.B. (2009). Bacterial effector binding to ribosomal protein S3 subverts NF-kappaB function. PLoS Pathog..

[7] Pham T.H., Gao X., Tsai K., Olsen R., Wan F., Hardwidge P.R. (2012). Functional differences and interactions between the *E. coli* type III secretion system effectors NleH1 and NleH2. Infect. Immun..

[8] Wan F., Weaver A., Gao X., Bern M., Hardwidge P.R., Lenardo M.J. (2011). IKKbeta phosphorylation regulates RPS3 nuclear translocation and NF-kappaB function during infection with *Escherichia coli* strain O157:H7. Nat. Immunol..

[9] Wan F., Anderson D.E., Barnitz R.A., Snow A., Bidere N., Zheng L., Hegde V., Lam L.T., Staudt L.M., Levens D. (2007). Ribosomal protein S3: A KH domain subunit in NF-kappaB complexes that mediates selective gene regulation. Cell.

[10] Khan M.A., Ma C., Knodler L.A., Valdez Y., Rosenberger C.M., Deng W., Finlay B.B., Vallance B.A. (2006). Toll-like receptor 4 contributes to colitis development but not to host defense during *Citrobacter rodentium* infection in mice. Infect. Immun..

[11] Ivison S.M., Himmel M.E., Hardenberg G., Wark P.A., Kifayet A., Levings M.K., Steiner T.S. (2010). TLR5 is not required for flagellin-mediated exacerbation of DSS colitis. Inflamm. Bowel Dis..

[12] Stecher B., Hardt W.D. (2008). The role of microbiota in infectious disease. Trends Microbiol..

[13] Round J.L., Mazmanian S.K. (2009). The gut microbiota shapes intestinal immune responses during health and disease. Nat. Rev. Immunol..

[14] Baumler A.J., Sperandio V. (2016). Interactions between the microbiota and pathogenic bacteria in the gut. Nature.

[15] Willing B.P., Vacharaksa A., Croxen M., Thanachayanont T., Finlay B.B. (2011). Altering host resistance to infections through microbial transplantation. PLoS ONE.

[16] Caporaso J.G., Lauber C.L., Walters W.A., Berg-Lyons D., Lozupone C.A., Turnbaugh P.J., Fierer N., Knight R. (2011). Global patterns of 16s rRNA diversity at a depth of millions of sequences per sample. Proc. Natl. Acad. Sci. USA.

[17] Morotomi M., Nagai F., Sakon H., Tanaka R. (2009). *Paraprevotella clara* gen. Nov., sp. Nov. And *Paraprevotella xylaniphila* sp. Nov., members of the family ‘*Prevotellaceae*’ isolated from human faeces. Int. J. Syst. Evol. Microbiol..

[18] Lucke K., Miehlke S., Jacobs E., Schuppler M. (2006). Prevalence of *Bacteroides* and *Prevotella* spp. In ulcerative colitis. J. Med. Microbiol..

[19] Ferreira R.B., Gill N., Willing B.P., Antunes L.C., Russell S.L., Croxen M.A., Finlay B.B. (2011). The intestinal microbiota plays a role in Salmonella-induced colitis independent of pathogen colonization. PLoS ONE.

[20] Schubert A.M., Sinani H., Schloss P.D. (2015). Antibiotic-induced alterations of the murine gut microbiota and subsequent effects on colonization resistance against *Clostridium difficile*. MBio.

[21] Ji Z.H., Ren W.Z., Gao W., Hao Y., Gao W., Chen J., Quan F.S., Hu J.P., Yuan B. (2017). Analyzing the innate immunity of NIH hairless mice and the impact of gut microbial polymorphisms on *Listeria monocytogenes* infection. Oncotarget.

[22] Hedberg M.E., Moore E.R., Svensson-Stadler L., Horstedt P., Baranov V., Hernell O., Wai S.N., Hammarstrom S., Hammarstrom M.L. (2012). Lachnoanaerobaculum gen. Nov., a new genus in the *Lachnospiraceae*: Characterization of *Lachnoanaerobaculum umeaense* gen. Nov., sp. Nov., isolated from the human small intestine, and *Lachnoanaerobaculum orale* sp. Nov., isolated from saliva, and reclassification of eubacterium saburreum (prevot 1966) holdeman and moore 1970 as *Lachnoanaerobaculum saburreum* comb. Nov. Int. J. Syst. Evol. Microbiol..

[23] Reeves A.E., Koenigsknecht M.J., Bergin I.L., Young V.B. (2012). Suppression of *Clostridium difficile* in the gastrointestinal tracts of germfree mice inoculated with a murine isolate from the family *Lachnospiraceae*. Infect. Immun..

[24] Antharam V.C., Li E.C., Ishmael A., Sharma A., Mai V., Rand K.H., Wang G.P. (2013). Intestinal dysbiosis and depletion of butyrogenic bacteria in *Clostridium difficile* infection and nosocomial diarrhea. J. Clin. Microbiol..

[25] Hart M.L., Meyer A., Johnson P.J., Ericsson A.C. (2015). Comparative evaluation of DNA extraction methods from feces of multiple host species for downstream next-generation sequencing. PLoS ONE.

[26] Hays M.P., Ericsson A.C., Yang Y., Hardwidge P.R. (2016). Vaccinating with conserved *Escherichia coli* antigens does not alter the mouse intestinal microbiome. BMC Res. Notes.

